# An Adaptive Traffic-Flow Management System with a Cooperative Transitional Maneuver for Vehicular Platoons

**DOI:** 10.3390/s23052481

**Published:** 2023-02-23

**Authors:** Lopamudra Hota, Biraja Prasad Nayak, Bibhudatta Sahoo, Peter H. J. Chong, Arun Kumar

**Affiliations:** 1 Department of Computer Science and Engineering, National Institute of Technology, Rourkela 769008, India; 2 Department of Electrical and Electronic Engineering, Auckland University of Technology, Auckland 1010, New Zealand

**Keywords:** platoon, traffic congestion, collision avoidance, CACC, merge maneuver, join maneuver, lane change

## Abstract

Globally, the increases in vehicle numbers, traffic congestion, and road accidents are serious issues. Autonomous vehicles (AVs) traveling in platoons provide innovative solutions for efficient traffic flow management, especially for congestion mitigation, thus reducing accidents. In recent years, platoon-based driving, also known as vehicle platoon, has emerged as an extensive research area. Vehicle platooning reduces travel time and increases road capacity by reducing the safety distance between vehicles. For connected and automated vehicles, cooperative adaptive cruise control (CACC) systems and platoon management systems play a significant role. Platoon vehicles can maintain a closer safety distance due to CACC systems, which are based on vehicle status data obtained through vehicular communications. This paper proposes an adaptive traffic flow and collision avoidance approach for vehicular platoons based on CACC. The proposed approach considers the creation and evolution of platoons to govern the traffic flow during congestion and avoid collisions in uncertain situations. Different obstructing scenarios are identified during travel, and solutions to these challenging situations are proposed. The merge and join maneuvers are performed to help the platoon’s steady movement. The simulation results show a significant improvement in traffic flow due to the mitigation of congestion using platooning, minimizing travel time, and avoiding collisions.

## 1. Introduction

**Figure 1:**
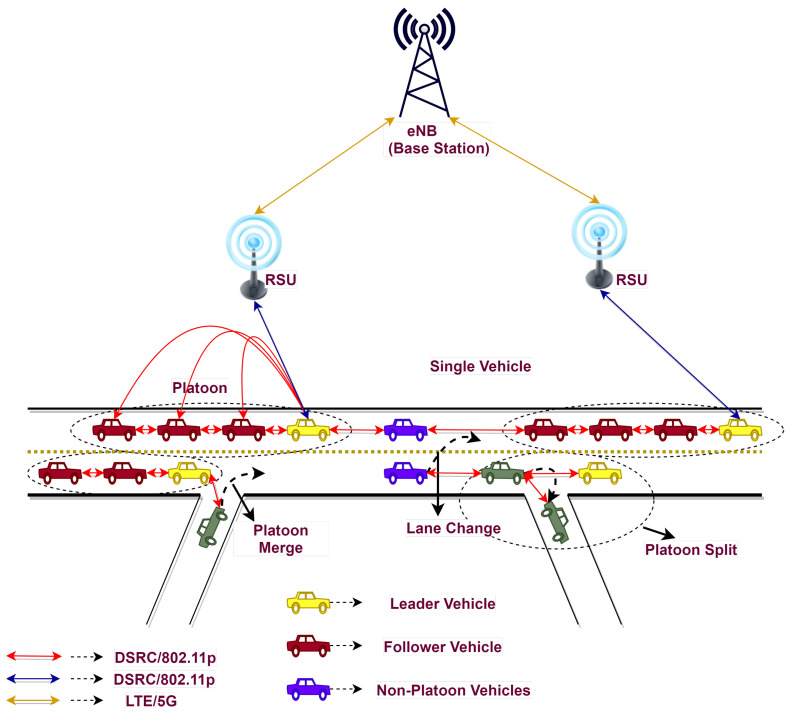
Platoon architecture.

The explosive growth in the number of vehicles has resulted in many critical social problems worldwide, such as road safety, traffic congestion, and fuel consumption. The high cost of construction and the lack of available land make road development unsustainable; even though it can somewhat decrease traffic congestion, it is not an efficient strategy. To deal with these issues, a platoon-based driving pattern, also known as a vehicle platoon, has received much attention in the past few years. A platoon consists of a vehicle which follows another vehicle and keeps a close and relatively constant safe distance from the one in front of it, termed cooperative driving [1]. Vehicle platooning is a technique where highway traffic is organized into groups of close-following vehicles called platoons [2]. Platooning enables vehicles to drive closer to others than regular vehicles at the same speed, which improves traffic throughput [3]. The communication can be V2V, i.e., inter-platoon, intra-platoon, and platoon to the non-platoon vehicle, and V2I, via RSU and base stations. Dedicated short range communication (DSRC) is an effective mode for short-range communication, whereas LTE/5G is found to be more efficient and reliable for long-range communication. Vehicle platooning is primarily used to alleviate traffic congestion and increase traffic flow in even dense scenarios. Figure 1 depicts a generalized platoon architecture consisting of the platoon and non-platoon vehicles with V2V and V2I communication.

The vehicles in the platoon coordinate among themselves by frequently exchanging periodic broadcast cooperative awareness messages (CAMs). The ETSI EN 302 637-2 standard specifies CAM-triggering conditions which depend on the dynamics of an originating vehicle [4]. The CAM is broadcast with a multi-channel access mechanism with a congestion control approach. The use of IEEE 802.11p in platooning is the subject of numerous studies and proposals, including message prioritization based on their type, dissemination, rate adaptation, and re-transmission [5]. An analysis of  connectivity probability within a platoon is presented in [6]. Still, there is a scope for the design of protocols for the delay-tolerant delivery of CAM in a platoon scenario and decentralized congestion control to avoid collision of CAM.

Cooperative driving can improve fuel economy, enhance infrastructure efficiency, and improve road safety [7,8]. Platooning implements the mutual assistance driving mechanism that aims to achieve semi-autonomous platooning by having a leader vehicle handled by either humans or an automation that drives a group of vehicles as followers. The onboard system uses the data from the leader and nearby vehicles via inter-vehicular communication (IVC) to manage the engine, brakes, and steering of followers, eliminating the need for the driver to steer manually, accelerate, or brake. Platooning involves lateral steering, coordinated acceleration, and braking via longitudinal collision mitigation [9], management protocols that monitor the creation of the platoon, driving maneuvers, and lane changing, thereby confirming that vehicle control is not the sole responsibility of either the human driver or automation to provide safety measures [10].

For performing basic maneuvers in the platoon, a lane is reserved for the vehicles traveling in the platoon. Each can perform several maneuvers to maintain the optimal size of the platoon and increase its efficiency. It becomes essential to differentiate between the platoon and non-platoon vehicles. Platoons in vehicular ad-hoc networks (VANet) depend very highly on effective communication between platoon members to carry out the basic platoon maneuvers such as platoon merge and join maneuvers. Failing to acquire efficient communication among the vehicles lead to the failure of the platoon. A platoon-enabled vehicle meets all of the functional requirements implemented for platoons. A non-platooned vehicle is driven manually or with the assistance of an automated cruise control (ACC) system [11]. In contrast, a platooned vehicle is a platoon member and can be either a leader or a follower.

Platooning is a viable solution as it eliminates frequent stopping and starting, which wastes time and fuel. Globally, the paradigm is shifting toward AVs, and the necessity arises to look for advanced long-term solutions to reduce congestion, travel time, and fuel consumption. The AVs’ penetration rate, defined as the ratio of AVs to total vehicles in a specified network, is predicted to increase from 24% to 87% by 2045 [12]. The massive increment of AVs in the future is the reason for platooning receiving serious attention in the past years. Because AVs are free of restrictions connected to driver behavior, such as reaction time and coordination, they could be one of the best possible platoon implementations. AVs with inter-vehicle communication (IVC) avoid traffic congestion, provide efficient coordination, and require a minimum response time by drivers, thereby avoiding accidents. Furthermore, analyzing safety features in scenarios such as crashes or accidents [13] is a critical consideration for AVs and human-driven vehicles. Current collision avoidance systems [14], such as ACC, adapt themselves using radar and lidar data, or communicate via breaking signals when delays or system failures could result in catastrophic damage. The proper routing of vehicles in the platoon is necessary to reduce traffic congestion. Therefore, the proposed approach’s primary objective is to minimize vehicle collision and travel time, leading to effective and reliable traffic-flow management.

### 1.1. Motivation

Traffic congestion and road safety are important issues of public safety worldwide. Annually, on average, approximately 150,000 people are killed, and 500,000 are injured in road accidents in India due to human errors such as distracted driving, over-speeding, and not complying with safety rules.Global statistics state that approximately 1.3 million people die yearly due to road traffic crashes. AVs moving in platoons are one of the best ways to reduce the accidents caused by human error and save passengers’ time by reducing traffic congestion. The World Health Organization (WHO), in November 2018, stated that the number of road traffic collisions has outreached the mark of 1.5 million per year. According to the 2008 World Health Statistics, road accidents were the 9th most significant cause of death, and at current rates, they will be the 5th leading cause of death by 2030 [15].

### 1.2. Contribution

The contributions of the proposed work are as follows:

1. This work proposes a mechanism to efficiently manage traffic during high congestion, thus reducing road fatalities.
2. The proposed work focuses on merging platoons into one platoon, improvising the traffic flow and reducing travel time.
3. Finally, traffic performance is enhanced by joining a single non-platooned vehicle into a vehicle platoon, and collision is reduced by lane-changing mechanisms.

The novelty of the proposed methodology lies in enhancing the overall management of traffic flow for AVs in a platoon. A structured traffic flow is achieved by configuring various algorithms such as maneuvering, re-routing, and lane-changing. In a real-time scenario, the platooning approach reduces the time to reach the destination and reduces the number of accidents as vehicles move synchronously.

### 1.3. Paper Organisation

The rest of the paper is organised as follows. Section 2 presents the related work, the proposed approach is demonstrated in Section 3, followed by simulations and results in Section 4. Finally, the conclusion and future work are presented in Section 5.

## 2. Related Works

Numerous research studies have been conducted to maintain traffic flow for vehicles in platoons in various aspects, such as leader vehicle failure [16], obstructions within the platoon, etc. [12]. Many researchers and automobile companies have proposed various techniques to increase AVs’ safety and road capacity. ACC, including no-communication functions, and CACC [17], including communication functions, are basic controller strategies followed for platoons. Platoons using CACC are fascinating because of their ability to increase road capacity in a very precise way [18].

A platoon-management protocol based on VANET and CACC vehicles which incorporate basic platooning maneuvers, such as platoon split, merge, and join maneuvers, is presented in [2]. However, this method has several disadvantages, such as a high delay during the merging of the platoon. An integrated ACC and CACC model with string stability in various traffic scenarios is implemented in [19]. Ploeg et al. [20] developed a hybrid controller for platoon merge and join maneuvers. The longitudinal control is handled by a continuous time system, while a discrete event supervisor decides the platoon merge-and-join maneuvers. However, where vehicle density is high, this technique suffers from frequent connection loss, resulting in a high packet-loss ratio. Huang et al. [21] demonstrated a cooperative platoon maneuver switching paradigm for merging and split maneuvers. However, this protocol causes significant latency and frequent connection loss. The authors in [22] proposed a distributed coordinated brake-control mechanism for longitudinal collision avoidance for multiple connected AVs in realistic scenarios. A brief statistical comparative study is conducted considering the initial velocity of the vehicle, inter-vehicular distance, communication topology, braking process, and different control mechanisms such as direct brake control, coordinated brake control, and driver reaction-based brake control.

The authors in [23] proposed an algorithm for platoon merge in cooperate driving that ensures effective platoon merging in all possible conditions. Wu et al. [24] proposed an adaptive velocity-based V2I fair access scheme based on IEEE 802.11, a distributive coordinate function for platoon vehicles. Roy et al. [25] proposed a model based on headway distribution of two-lane roads under different traffic situations with varying distributions such as Poisson, log-logistic and Pearson. In [26], authors investigated an event-based control and scheduling co-design technique for a platoon with packet disorder and communication delay, reducing congestion and stabilizing the system. Nevigato et al. [27] provide a collision-avoidance solution in a mobile edge computing-based environment to avoid accidents.

Hu et al. [28] proposed a reliable, trustworthy platoon-based service recommendation technique to help the user eliminate malicious platoons using V2V communication. Zhang et al. [29] present a trust-based and privacy-preserving platoon approach to enable the user vehicle to avoid the malicious leader vehicle. However, the method suffers from a problem in accurately identifying the trust value. The author in [30] proposed a method to distribute urban platooning towards high flexibility, adaptability and stability to solve the problem of vehicle density in urban areas, traffic lights etc. This method suffers from communication failure in dense vehicle scenarios.

The authors in [31] have argued that platoon-based driving offers a plethora of benefits. Firstly, since vehicles in the same platoon are substantially closer to one another, the traffic congestion may be reduced, and the road capacity is increased appropriately. Secondly, the platoon structure can significantly decrease energy use and exhaust emissions, as a platoon’s vehicles can be streamlined to reduce air resistance. Third, driving has become safer due to contemporary technologies; it becomes more secure and comfortable in a platoon. Lastly, platoon-based data dissemination mechanisms are efficient due to their position and synchronization, significantly enhancing vehicular network performance.

Segata et al. [32] developed an integrated simulator as a novel contribution towards research in platooning techniques. This is the first attempt to describe a high-level platoon management protocol in VANET-enabled vehicles that employ wireless V2V and V2I communication with IEEE 802.11p. Moreover, there is scope to explore the topic in-depth, and more simulation situations can be tested utilizing this simulator for vehicular platooning.

In this paper, three scenarios (join maneuver, merge maneuver, and lane change) are implemented, and the behavior of their speed, acceleration, and distance are analyzed. Taking different aspects from the literature, a meticulous solution is designed for AVs moving in platoons. Further, the total time to reach the destination for a platoon, non-platoon, or obstruction within the platoon (i.e., lane change or failure of leader vehicle) and multiple platoon scenarios (i.e., merge maneuvers) are explored.

## 3. Proposed Work

This section describes the system model, assumptions, and the methodology used for the proposed work to manage traffic flow using platooning.

### 3.1. System Model

Each vehicle is equipped with sensors and a GPS, and uses wireless access in vehicular environment (WAVE) as the inter-vehicle communication protocol based on DSRC. The onboard unit is capable of localization and time synchronization due to the equipped GPS. To exchange information about vehicle dynamics and emergency data, each vehicle in the platoon periodically transmits beacons. The vehicle movement is based on CACC, and thus, the cooperative movement considerably reduces the inter-vehicle spacing within a platoon. The intra-platoon communication must be relayed when the platoon is too large. To ensure safety, the inter-platoon separation is greater than the intra-platoon spacing. The size of the platoon is constrained to allow direct communication between platoon members inside the same platoon via one-hop communication. During the evaluation, the platoon’s topology is considered to be static. This leads to proper channel utilization with a reduced synchronization overhead. In a real-world situation, there is a possibility of more than one platoon driving in the same lane, here, we have considered *N*-platoon. The  $j^{th}$ platoon is designated as $P_{j}$. The $i^{th}$ vehicle within $j^{th}$ platoon is denoted as $V_{j,i}$, where *i* = 1…*N* and *j* = 1…*M*. The communication only takes place between the last vehicle of leading platoon and first vehicle of the following platoon. This reduces the interference between non-neighboring platoons.

### 3.2. Assumptions

1. All vehicles on the road are AVs to make communication reliable and compatible; there are no human-driven vehicles.
2. Let $d_{1}$, $d_{2}$, $d_{3}$ be the current density, threshold density, and normal density, respectively. The density of the AV represents the number of AVs per unit length-segment of the lane. AVs are generated by Poisson distribution with arrival rate λ as $V_{i}$, where *i* = 1, 2, 3, …, *N*.
3. The initial route and alternate routes are generated against each source destination. The source and destination of each AV are assumed to be known, creating platoons *P* having a minimum of four AVs, where *P* = $V_{1}$, $V_{2}$, $V_{3}$, …, $V_{N}$. The set of AVs fetched in each platoon can be stated as $P_{j}$$V_{i}$ where *j* = 1, 2, 3, …, *M*, for example, $P_{1}V_{4}$, $P_{1}V_{5}$, $P_{2}V_{6}$, $P_{2}V_{4}$, $P_{3}V_{4}$, $P_{3}V_{8}$, and so on.
4. The speed of the AV, acceleration, minimum gap, and distance to the leader are assumed to be known. The AVs in the platoons are induced to proceed from the source towards the destination, following the leader AV. The platoon vehicles move in a dedicated lane of the four-way highway. This mechanism minimizes the hindrance of human-driven vehicles in the other lanes.
5. The AVs broadcast CAM via a dedicated channel (CCH) at a frequency of 10 Hz, as per 802.11p specification. As an example, standard single-radio transceivers for platooned AVs are considered which are continually modulated to the CCH to broadcast and receive CAM [33]. The information about the density of AVs, speed, acceleration, and flow of AVs individually and in the platoon are utilized for congestion detection and avoidance during rerouting.
6. The car-following mobility model is similar to the one used in the PLEXE simulator i.e., the CACC approach. The CACC approach exploits the communication among vehicles via IVC. The control law for the CACC model considered for our implementation is based on the theory of consensus [32].

### 3.3. Proposed Methodology

The proposed algorithm (Algorithm 1) assumes that all the vehicles on the lanes are autonomous, and that there are no cooperation or communications glitches among the AVs. The AVs are assumed to know each other’s location and speed details. Platoons of different sizes are considered to travel in highway lanes. Initially, the source and destination of the AVs and the current and alternate routes are assumed to be known. The AVs with the same destination are in one platoon according to the proposed algorithm. After the formation of all the platoons, the densities of the platoons are analyzed. Based on the platoon density, it is decided whether to keep the platoon on the same route or reroute. Let *d* be the total density of AVs in a road length stated as AVs per unit road length. The current density $d_{1}$, which is the maximum density for free flow traffic in the platoon at a particular instance of time, and the threshold density $d_{2}$, which is the maximum density for a road length, are the two basic types of densities considered in traffic-flow management. The total number of AVs generated by the free flow mobility model per unit length of the road is denoted by $d_{3}$. The distance between two AVs is inversely proportional to the density of the AVs in that particular lane. The free-flow model is designed with reference to [34], taking $d_{1}$ to be 20 and $d_{2}$ to be 50 for a single lane.

**Figure 2:**
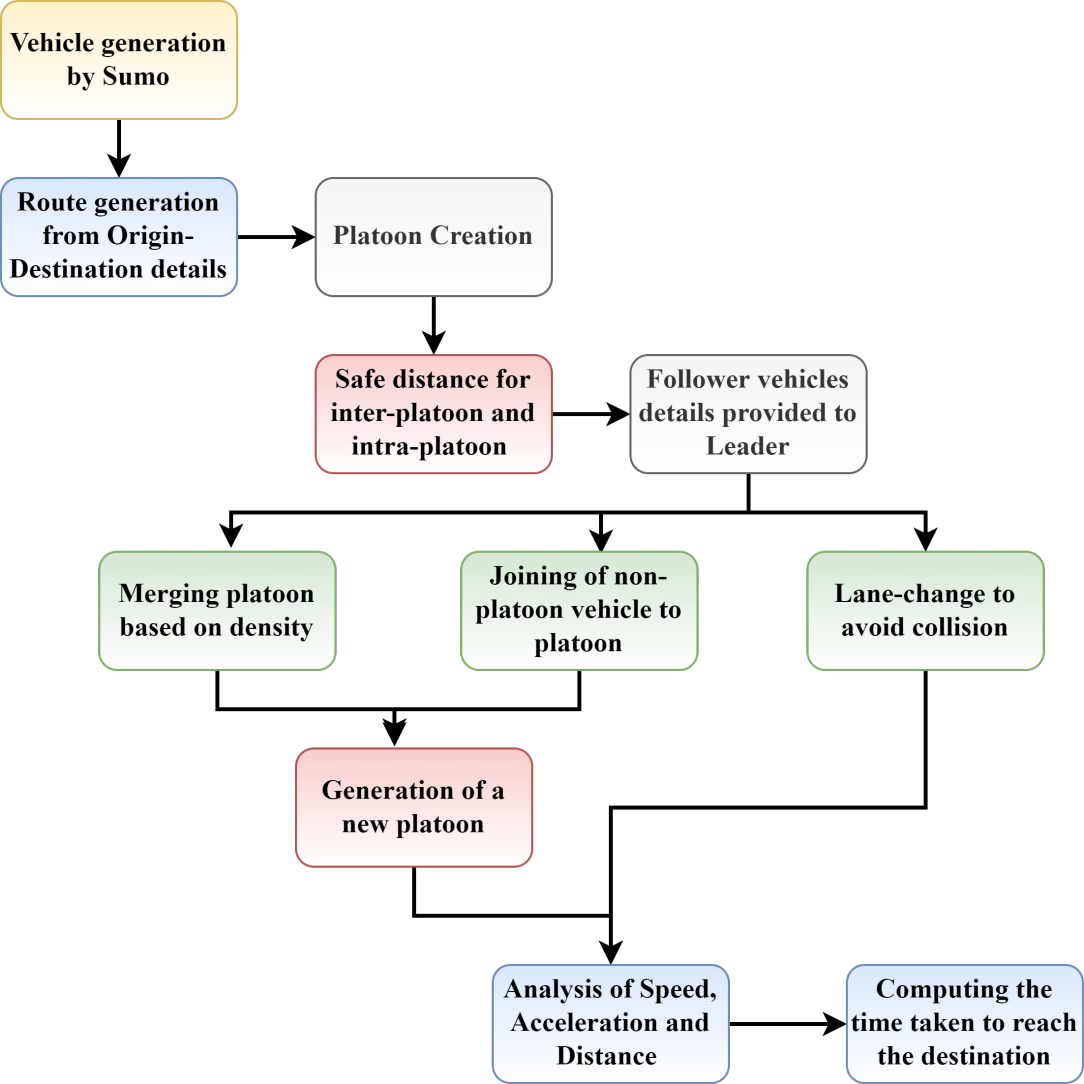
Flow diagram of proposed model.

Apart from density, the time delay of platoons is checked and compared with the threshold value. If the delay time is greater than the threshold value, then the route of the platoon is changed (re-routing). If the delay is less than or equal to the threshold value, the same path is followed. Sometimes, it may be possible that the platoon is not at its best efficiency due to the presence of many smaller platoons on the road, which leads to many leaders. Therefore, to improve the algorithm’s efficiency, two smaller platoons are merged into one larger platoon, resulting in one leader. In addition, it may be possible that instead of merging platoons, a single AV is added to the platoon. Therefore, the joining maneuver is performed. During this maneuver, the AVs in a platoon increase or decrease their speed synchronously. When an obstruction occurs between the platoon to eliminate these situations, a collision-avoidance mechanism is also implemented. The mechanism will reroute the platoons in the lane to another lane if it is blocked/obstructed. At the end of the journey, if all platoon AVs reach the destination safely, the platoon is marked as “Successfully Reached”. Figure 2 presents the gist of the approach for analyzing platooning maneuvers. Table 1 shows the summary of the notation used in the algorithm.
**Algorithm 1** An algorithm for traffic management using platooning  1:Start  2:Set $d_{1}$, $d_{2}$, $d_{3}$;  3:for each $P_{j}$ do  4:**if** ($d_{1}$ >= $d_{2}$) **then**  5:     Platoon Rerouting  6:**else if** ($d_{1}$ <= $d_{3}$) **then**  7:     No Rerouting  8:**end if**  9:for each $P_{j}$ following $P_{1}$ do10:**if** ( $T_{d}$ > $Th_{d}$) **then**11:     Platoon Rerouting12:**else if** ($T_{d}$ < $Th_{d}$) **then**13:    No Rerouting14:**end if**15:**if** ($P_{1}$ receives merge request from $P_{2}$) **then**16:    **if** ($P_{1}$, *$P_{2}$ϵ Same Lane)* **then**17:        **if** ((size of $P_{1}$ + size of $P_{2}$) <= $Th_{den}$) **then**18:            Merge $P_{1}$ and $P_{2}$19:        **end if**20:    **end if**21:**end if**22:**if** ( $P_{j}$ receives join request from $V_{i}$) **then**23:    **if** ($P_{j}$, *$V_{i}$ϵ Same Lane)* **then**24:        **if** ((size of $P_{j}$) < $Th_{den}$) **then**25:           Join $V_{i}$ into $P_{j}$26:        **end if**27:    **end if**28:**end if**29:**if** ($P_{j}$L==inactive) **then**30:  Assign new Leader to $P_{j}$31:**end if**32:**if** (Obstruction between $P_{j}$) **then**33: Split $P_{j}$ in $P_{j}$ and $P_{j}$′ and assign L′ to $P_{j}$′34:**end if**35:for each $P_{j}$ do36:**if** (collision) **then**37:     lane change38:**end if**39:End

## 4. Simulation and Results

This section presents the various scenarios and an analysis of results in detail.

### 4.1. Simulation Tool

This section describes the simulation tool used for the proposed approach. Simulation is performed on PLEXE, which supports vehicle platooning, based on network simulation platforms OMNeT++, VEINs, and simulation of urban mobility (SUMO). SUMO is an open-source traffic simulator used to optimize traffic signals, investigate route choice and forecast traffic. VEINS and OMNeT++ handle V2V and V2I communications. VEINs are used as a vehicle communication technology. Its broad class of libraries improve the realism and efficiency of traffic simulations. VEINs and OMNeT++ provide several capabilities that allow vehicles to communicate with one another. The VEINs framework communicates vehicles via the IEEE 802.11p and 1609.4 DSRC/wireless access.

### 4.2. Simulation Parameters

This section presents the simulation parameters. Table 2 shows the different parameters used in the simulation.

### 4.3. Merge Maneuver (Scenario 1)

Several maneuvers are performed for merging, joining, and collision avoidance. This section presents the results and discussions. In this section, the AVs are represented as cars in the figures.

**Figure 3:**
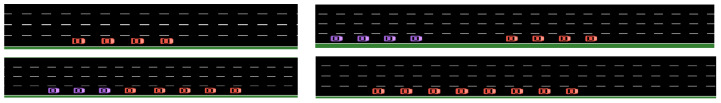
Merging of platoons.

The scenario taken for merging platoons is depicted in Figure 3. Two or more platoons moving in the same lane and joining to form a single large platoon is a merging maneuver [35]. Whenever the platoon size is smaller than the optimal size, a merge maneuver is initiated. We assume two platoons Y and X, where Y is the rear and X is the front platoon, with one platoon leader and three followers. The optimal size of the platoon is taken to be 8. Initially, the leader of platoon X receives the merge request from the leader of platoon Y. The X-platoon leader can either accept or decline the merge request based on several factors, such as the capacity of the platoon.

On receiving approval for merging from the leader of X, the leader of Y reduces its intra-platoon-spacing [36] by increasing its speed to catch up with the front platoon. After the leader of platoon Y is in tune with the front platoon X, Y’s leader sends a request to all of the platoon’s members for leader change. After changing the leader, all the follower AVs start communicating with the platoon leader, and finally, the rear platoon leader becomes a follower of platoon X.

**Figure 4:**
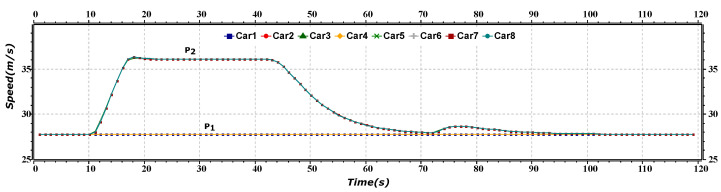
Speed versus time graph of merge maneuver.

Figure 4 represents the speed versus time of merge maneuver of the platoon. The $P_{1}$ (including Car1–Car4) and the $P_{2}$ (including Car5–Car8) line shows the speed of the front and rear platoons, respectively. Initially, both the platoons are moving at the same speed, and after approximately 12 s, the rear platoon increases its speed and then moves constantly for a few seconds. After reaching a time period of nearly 45 s, the rear platoon starts reducing its speed to merge into the first platoon. After merging, the platoon’s AVs move at a constant speed agaiuntil the destination.

**Figure 5:**
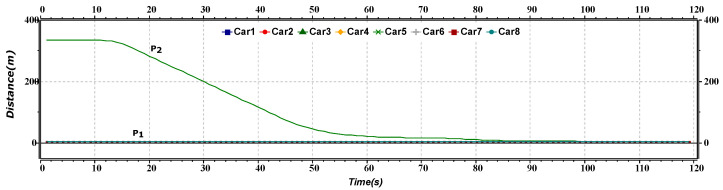
Distance versus time graph of merge maneuver.

Figure 5 represents the distance versus time of the merge maneuver of the platoon. The graph shows the distance between both the platoons. Initially, both the platoons have an inter-platoon distance of approximately 330 m. After approximately 12 s, as the rear platoon leader increases its speed, and the distance between the platoon starts decreasing. After nearly 80 s, all the AVs have a constant distance between them as both platoons merge to form one platoon.

**Figure 6:**
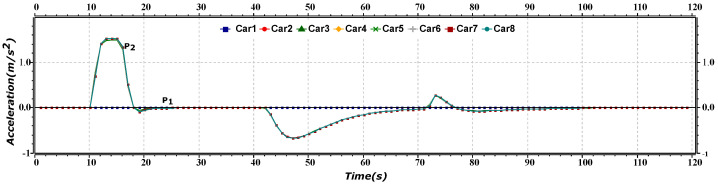
Acceleration versus time graph of merge maneuver.

Figure 6 represents the acceleration versus the time of the merge maneuver of the platoon. The $P_{1}$ (including Car1–Car4) line shows the acceleration of the front platoon, whereas the $P_{2}$ (including Car5–Car8) shows the acceleration of the rear platoon. Initially, both platoons are moving with the same acceleration. After approximately 12 s, as the rear platoon increases its speed, its acceleration changes from 0 to 1.5 m/s^2^. After nearly 43 s, acceleration starts reducing as the rear leader AV decreases its speed to match the speed of the front platoon AV.

### 4.4. Join Maneuver (Scenario 2)

**Figure 7:**
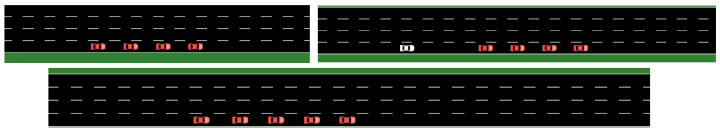
Joining maneuver.

The scenario of joining platoons is depicted in Figure 7.

In the joining maneuver scenario, a single platoon of four AVs traveling on the freeway is taken, while a fifth AV requests the platoon leader to join at the tail of the platoon. The non-platooned AV asks the platoon’s leader to join the platoon and waits for a response. The leader accepts the request of the fifth AV (Car5) and responds with a message that includes essential information about the platoon, such as lane number and joining position, and waits for the joiner to become closer. The joiner AV uses that information to come close to the platoon’s last AV, increasing its speed and managing the distance. When a joiner AV reaches a predetermined distance from the platoon’s last AV, it signals to the leader that it is ready to join.

**Figure 8:**
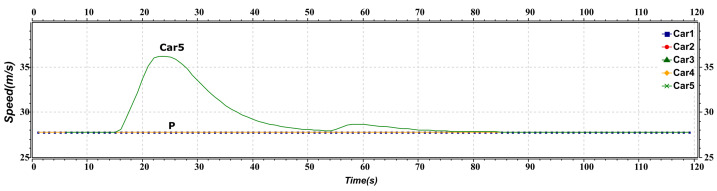
Speed versus time graph of joining maneuver.

Figure 8 represents the speed versus time of the joining maneuver of the platoon and AV. $P_{1}$ (including Car1–Car4) shows the speed of the front platoon, whereas the Car5 line indicates the speed of the non-platooned AV. Initially, both are moving at the same speed. At 17 s, the non-platooned AV increases speed and constantly moves until the time reaches 23 s. After reaching 32 s, the AV starts reducing its speed to merge into the platoon. After joining, the AV becomes part of the platoon and starts moving at a constant speed.

**Figure 9:**
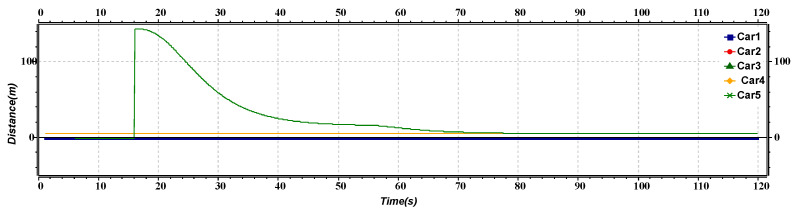
Distance versus time graph of joining maneuver.

Figure 9 represents the distance versus time of the joining maneuver of the platoon and AV. The graph shows the distance between the platoon and the AV. After approximately 16 s, the distance between the platoon and the AVs decreases as the AV increases its speed. After reaching a time of nearly 50 s, all the AVs have a constant distance between them as AVs merge into the platoon merge to form one platoon.

**Figure 10:**
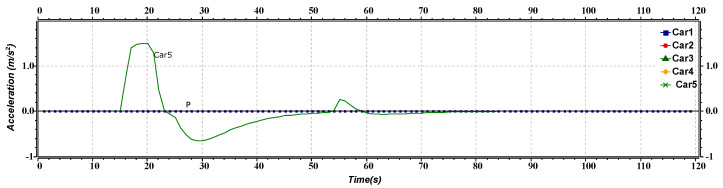
Acceleration versus time graph of joining maneuver.

Figure 10 represents the acceleration versus time of joining maneuver of the platoon and AV. The $P_{1}$ shows the speed of the front platoon, whereas the Car5 line shows the AV’s speed. Initially, both are moving at the same speed. After approximately 16 s, the AV increases speed and then reduces at 22 s. After reaching a time of nearly 32 s, the AV begins reducing its speed to match the speed of the first platoon. As the AV is entirely ready to merge into the platoon, it needs to adjust its speed, so at approximately 45 s, its acceleration shows some fluctuations. After merging, the AVs of the platoon move at a constant speed.

### 4.5. Collision-Avoidance (Scenario 3)

**Figure 11:**
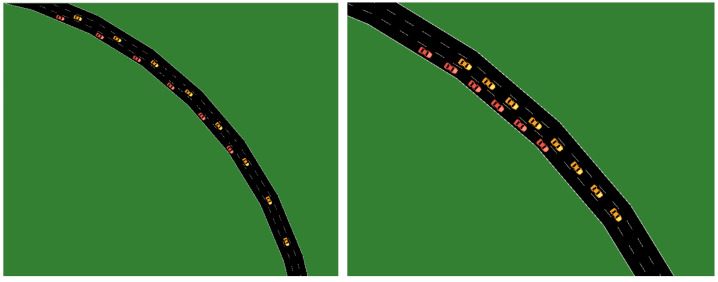
Collision avoidance by lane change.

This section focuses on avoiding collision by changing the lane of the whole platoon whenever any obstruction occurs during the journey. Figure 11 shows two platoons with 6 and 8 AVs moving in different lanes. The figure depicts collision avoidance due to obstruction by lane change, where the platoon of AVs with 6 AVs moves to the alternate lane.

**Figure 12:**
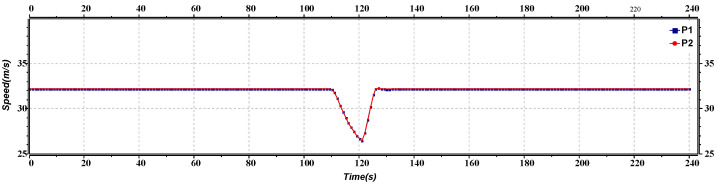
Speed versus time graph of lane change.

Figure 12 represents the speed versus the time of lane change of the platoon leaders. Initially, both the platoons are moving at the same speed. After approximately 50 s and 90 s, the second platoon needs to change the lane to avoid the collision, decreasing its speed to move to another lane and then moving with the same speed as earlier. After approximately 90 s, as the first platoon also needs to change lanes to avoid the collision, it also decreases its speed to move to another lane and then moves with the same speed as earlier.

**Figure 13:**
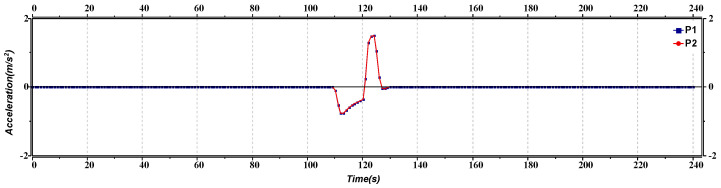
Acceleration versus time graph of lane change.

Figure 13 represents the platoon leaders’ acceleration versus the time of lane change. Initially, both the platoons are moving with the same acceleration. After approximately 50 s and 90 s, as the second platoon reduces its speed for a lane change, its acceleration varies from 0 to −1.5 m/s^2^ every time. The first platoon also needs to change lane to avoid a collision; it adjusts its speed to approximately 90 s, and its acceleration shows some fluctuations due to the lane change.

**Figure 14:**
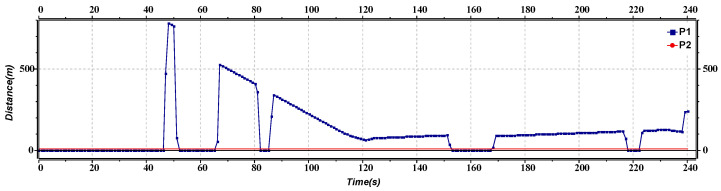
Distance versus time graph of lane change.

Figure 14 represents the distance versus the time of inter-platoon lane change, specifically between the leader of both platoons. Initially, the distance between the platoon leaders is constant. As the lane change is about to occur, the inter-platoon distance deviates, with the first deviation occurring at around 30 s. The graph predicts that it is challenging to maintain a constant distance between platoons during the lane change, but a safe distance is still maintained. This provides a research scope to formulate a critical safe distance model during the lane change, thus avoiding crashes. The proposed algorithm has focused on the safe distance between inter and intra-platoon to some extent which can be considered in future work.

### 4.6. Comparison of All Scenarios

This section describes a comparative analysis of the number of AVs versus the total time to reach the destination in different scenarios with and without platoons, lane change, leader failure, and merge maneuver. For a comparative analysis of the total time taken to reach the destination for 500 AVs, these AVs were randomly generated by the free flow model in veh/hr, and their travelling time is computed.

The time an AV takes to get from its starting point to its destination is known as the travel time. AVs on highways move at a reasonable speed (90 to 140 km/h) to consume the least amount of fuel. A successful platooning mechanism also reduces fuel consumption due to the reduced travel time. In our simulation environment, the traveling time is defined as the interval between the AV’s generation (entry of lane) and the moment it reaches its destination point. Each AV keeps track of its generation time. When it reaches its destination, it uses the time difference to calculate the distance traveled and time taken, based on relative speed.

Every AV predicts the time it will take to reach its destination when it merges onto the freeway, assuming a constant relative speed with the leader. AVs calculate the journey time by recording their actual travel time after arriving. This statistic allows us to demonstrate how platooning affects travel time. In this study, the platoon leader determines the speed of platoon members. The platoon maneuvers reduce the travel time to a great extent, contributing towards fuel-consumption minimization.

**Figure 15:**
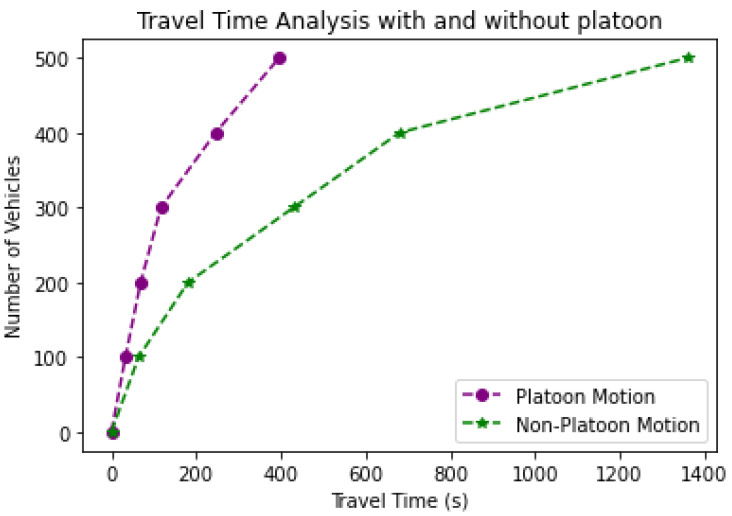
Comparison of platoon and non-platoon scenarios in terms of total time taken to reach destination.

Figure 15 compares AV’s movements with and without platooning, computing their travel time. The simulated results show that the traveling time of 500 AVs is 1360 s (nearly 23 min) without platooning. For a platoon scenario where the AVs moved in a platoon with cooperative speed and time-headway and rerouted to an alternate path in case of collision, the traveling time is 396 s (nearly 7 min). Therefore, we can conclude that the travel time is minimized by approximately 69% in a platoon scenario.

**Figure 16:**
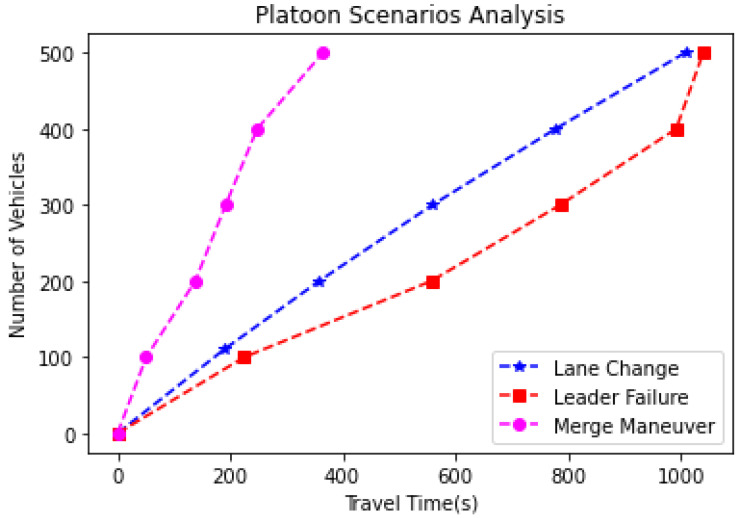
Number of AVs vs travel time to reach destination in different scenarios.

In Figure 16, three scenarios are analyzed. The graph depicts the total traveling time taken by 500 AVs to reach their destinations. The three analyses include the scenario of lane change due to obstruction or chance of collision, the scenario when the leader AV fails, and the scenario with multiple platoons, i.e., merge maneuver. The results show that it takes 1008s (nearly 16 min) for 500 AVs to reach their destination when the lane change is performed. Identifying and choosing a new leader if the leader fails is time-consuming in case of any platoon interruptions. When the leader fails, it takes almost 1040 s (nearly 17 min) for 500 AVs to reach their destination. The travel time in case of leader AV failure is more than the travel time in a lane change. Finally, when small platoons merge into one large platoon, as in the merging maneuver, it significantly improves the AV platooning performance. It takes almost 365 s (nearly 6 min) for 500 AVs to arrive at the destinations. Thus, based on these results, it can be observed that merging platoons into one platoon could improve the traffic flow by reducing the travel time by 64%. However, in a real-time scenario, there will be a trade-off between travel time and platoon size, as a very long platoon can tend to congestion of road traffic. This analysis provides scope for designing an optimized algorithm considering the number of AVs, platoon size, and travel time, thereby monitoring fuel consumption.

## 5. Conclusions and Future Works

This paper has presented an adaptive traffic-flow management mechanism with collision avoidance for vehicular platoons. The proposed model has contributed toward traffic-flow management of AVs using platooning by rerouting platoons in a collision situation, thereby avoiding traffic congestion. The merge and join maneuvers are performed to reduce the total travel time and road overhead by merging two small platoons into a single platoon. The lane-change mechanism of the platoon has been implemented to avoid vehicular collisions, thereby reducing the time to reach the destination. The comparative study shows that merging small platoons can minimize traveling time in a platoon scenario. Thus, this study focuses on optimizing and analyzing travelling delay for platoon scenarios.

In future, leaving and splitting maneuvers will be implemented for different sections of the platoon. A headway control will be designed for platoon modeling to minimize rear-end and side crashes. 

## Figures and Tables

**Table 1:** Summary of notations.

Notations	Description
$d_{1}$	Current Density of AVs
$d_{2}$	Threshold Density of AVs
$d_{3}$	Normal Density of As
$T_{d}$	Time Delay
$Th_{d}$	Threshold Value of Delay
$Th_{den}$	Threshold Density of Platoon
$P_{j}$	Identity of the $j^{th}$ platoon
$V_{i}$	Identity of the $i^{th}$ AV
*L*	Lane number

**Table 2:** Simulation Parameters.

Parameter	Values
AV’s length	4 m
Optimal platoon size	8
Controller	ACC, CACC
Leader headway	1.2 s
Maximum speed (leader)	33.34 m/s
Maximum acceleration	$3\;m/s^{2}$
Maximum deceleration	$3\;m/s^{2}$
Lanes	4
Platoon size	4,6,8
Simulation rime	Merge-and-join maneuver (120 s), lane change (300 s)
PHY/MAC Model	IEEE 802.11p
MAC Model	1609.4

## Data Availability

Not applicable.
